# *Mangifera indica* L., By-Products, and Mangiferin on Cardio-Metabolic and Other Health Conditions: A Systematic Review

**DOI:** 10.3390/life13122270

**Published:** 2023-11-28

**Authors:** Giulia Minniti, Lucas Fornari Laurindo, Nathalia Mendes Machado, Lidiane Gonsalves Duarte, Elen Landgraf Guiguer, Adriano Cressoni Araujo, Jefferson Aparecido Dias, Caroline Barbalho Lamas, Yandra Crevelin Nunes, Marcelo Dib Bechara, Edgar Baldi Júnior, Fabrício Bertoli Gimenes, Sandra Maria Barbalho

**Affiliations:** 1 Department of Biochemistry and Pharmacology, School of Medicine, Universidade de Marília (UNIMAR), Marília 17525-902, SP, Brazil; giulia.minniti@hotmail.com (G.M.); nathaliamendesmachado@gmail.com (N.M.M.); elguiguer@gmail.com (E.L.G.); adrianocressoniaraujo@yahoo.com.br (A.C.A.); dib.marcelo1@gmail.com (M.D.B.); 2 Department of Biochemistry and Pharmacology, School of Medicine, Faculdade de Medicina de Marília (FAMEMA), Marília 17519-030, SP, Brazil; yandracrevelin@hotmail.com; 3 Department of Biochemistry and Nutrition, School of Food and Technology of Marília (FATEC), Marília 17500-000, SP, Brazil; lidianegonsalves.duarte@fate.sp.gov.br (L.G.D.); jeffersondias@unimar.br (J.A.D.); reumatoedgar@hotmail.com (E.B.J.); fabricio.bg@gmail.com (F.B.G.); 4 Postgraduate Program in Structural and Functional Interactions in Rehabilitation, School of Medicine, Universidade de Marília (UNIMAR), Marília 17525-902, SP, Brazil; 5 Department of Gerontology, School of Gerontology, Universidade Federal de São Carlos (UFSCar), São Carlos 13565-905, SP, Brazil; carolblamas@gmail.com

**Keywords:** *Mangifera indica* L., by-products, antioxidant, anti-inflammatory, diabetes, obesity, cardiovascular disease

## Abstract

Mango and its by-products have traditional medicinal uses. They contain diverse bioactive compounds offering numerous health benefits, including cardioprotective and metabolic properties. This study aimed to explore the impact of mango fruit and its by-products on human health, emphasizing its metabolic syndrome components. PUBMED, EMBASE, COCHRANE, and GOOGLE SCHOLAR were searched following PRISMA guidelines, and the COCHRANE handbook was utilized to assess bias risks. In vivo and in vitro studies have shown several benefits of mango and its by-products. For this systematic review, 13 studies met the inclusion criteria. The collective findings indicated that the utilization of mango in various forms—ranging from fresh mango slices and mango puree to mango by-products, mango leaf extract, fruit powder, and mangiferin—yielded many favorable effects. These encompassed enhancements in glycemic control and improvements in plasma lipid profiles. Additionally, mango reduces food intake, elevates mood scores, augments physical performance during exercise, improves endothelial function, and decreases the incidence of respiratory tract infections. Utilizing mango by-products supports the demand for healthier products. This approach also aids in environmental conservation. Furthermore, the development of mango-derived nanomedicines aligns with sustainable goals and offers innovative solutions for healthcare challenges whilst being environmentally conscious.

## 1. Introduction

*Mangifera indica* L., popularly known as mango, is one of the most common tropical fruits of the genus Mangifera, which comprises around 30 species of fruit trees in the *Anacardiaceae* family. It originates from Malaysia and India, has been domesticated and cultivated for more than 4000 years, and is produced in more than 100 countries, including Pakistan, China, Philippines, Thailand, Nigeria, Israel, Italy, Spain, Mexico, and Brazil, with India as the world’s largest producer. Due to its enormous popularity, pleasant flavor, and excellent nutritional value, it ranks fifth in production among perennial fruit trees worldwide and second among the most commercialized tropical fruits, with a production of more than 40 million tons in 2021 [1,2,3].

**Figure 1:**
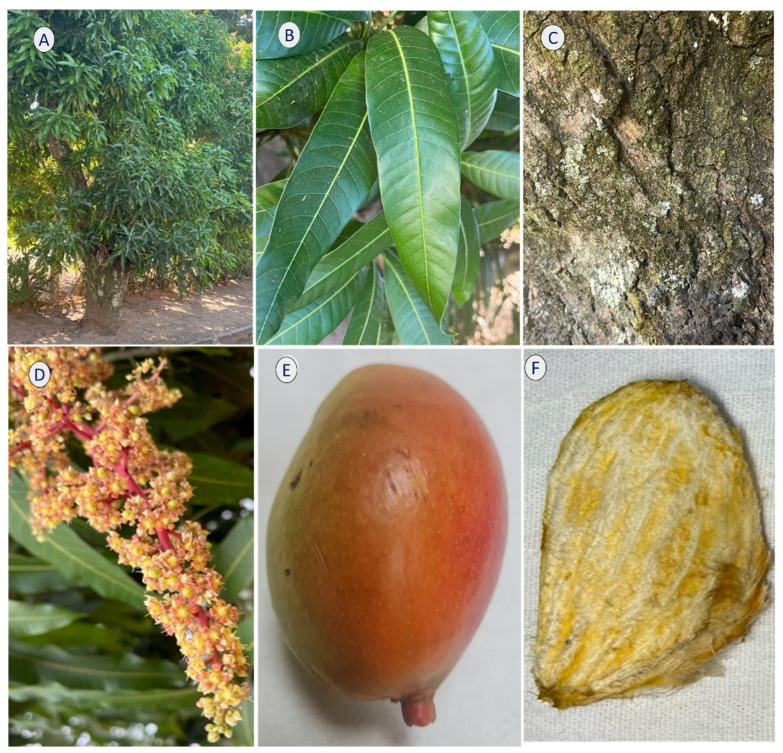
Mango tree. (**A**) tree, (**B**) leaves, (**C**) bark, (**D**) flowers, (**E**) fruit, and (**F**) seed.

Various parts of the mango, such as its fruits, flowers, leaves, roots, and peels, have been commonly used to treat multiple diseases. Its fruits are also rich in vitamin C and amino acids. The fruits, leaves, peels, and seeds are rich in phytochemicals, including polyphenols, terpenoids, carotenoids, and phytosterols. These bioactive compounds provide several health benefits, including anti-inflammatory, immunomodulatory, antibacterial, antiviral, antifungal, and anticancer effects [2,3,4,5,6,7]. Figure 1 shows the main parts of the tree.

Mangoes contain ascorbic acid, gallic acid, protocatechuic acid, chlorogenic acid, and vanillic acid as the major phenolics. Mango pulp contains sugars; vitamins (such as ascorbic acid and carotenoids); polyphenols such as xanthonoids (mangiferin); flavonoids such as catechins, kaempferol, rhamnetin, quercetin, and anthocyanins; tannins such as gallotannins; and phenolic acids and derivatives thereof, such as ellagic acid, gallic acid, protocatechuic acid, methyl gallate, and propyl gallate [8].

Among the most significant phenolic compounds contained in mango peels, mangiferin (1,36,7-tetrahydroxyxanthone-C2-β-D-glucoside) is found in various amounts and forms. Although the primary source of this phenolic compound is *Mangifera indica*, it is also found in other species (*Anacardiaceae*, *Gentianaceae*, and *Iridaceae* families). Mangiferin has been investigated for its potential as a health promotion agent. It can exhibit antioxidant, anti-inflammatory, immunomodulatory, antibacterial, and anti-obesity effects. Due to these effects, it is a promising adjuvant therapeutic to chronic disorders such as cardiovascular, renal, and pulmonary diseases; neurodegenerative disorders; obesity; diabetes; and metabolic syndrome [2,9,10,11]. Table 1 shows the main bioactive compounds of mango and its by-products.

Besides its consumption *in natura*, mango is used to prepare many food products such as jellies, liqueurs, juice, nectar, and vinegar. Furthermore, it is used in the pharmaceutical and cosmetic industries to produce herbal medicines and cosmetics [12,13]. This vast range of uses leads to a substantial economic and environmental impact regarding the generation of by-products, including leaves, peel, seeds, bark, and extracts (from leaves, peel, and bark) [14,15,16]. 

These by-products exhibit several bioactive compounds beneficial to human health, including vitamins (A, B, C, and E) and other antioxidants such as mangiferin, benzophenones (iriflophenone 3-C-glucoside), anthocyanins, phenolic acids, gallic acid, coumarin, quercetin, and flavonoids [12,17,18,19,20,21,22,23,24].

Since mango and its by-products have a lot of versatility in terms of the presence of bioactive compounds and can therefore contribute to the prevention and treatment of health conditions, the aim of this study is to investigate the effects of mango, mangiferin, and its by-products on cardiometabolic and other health conditions. Only two other systematic reviews have investigated the impact of mango on human health. Zarasvand et al. [25] investigated the effects of the mango plant on type 2 diabetes mellitus; however, they did not investigate the impact on metabolic syndrome, a condition closely related to hyperglycemia. Lum et al. [26] investigated the effects of mangiferin only on memory impairment. For this reason, and to the best of our knowledge, this is the first systematic review addressing the effects of mango, mangiferin, and by-products on human health, with an emphasis on metabolic syndrome, which is a risk condition for the development of cardiovascular diseases, which are among the leading causes of death in the world.

**Table 1:** Main bioactive compounds of *M. indica* and their effects on human health.

Phytochemical	Structure	Actions	Ref.
Mangiferin	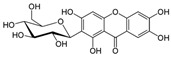	Antidiabetic, hypolipidemic, antioxidant, and anti-inflammatory	[17,18]
Catechin	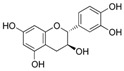	Antidiabetic, antioxidant, antimicrobial, and anti-inflammatory	[18,27,28]
Quercetin	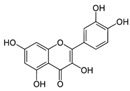	Antioxidant and anti-inflammatory	[28]
Ferulic acid	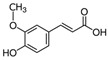	Antioxidant, anti-inflammatory, and photoprotective	[18,28]
Vanillic acid	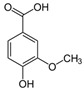	Antioxidant	[18,29]
4-hydroxybenzoic acid	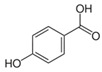	Antioxidant and anti-inflammatory	[28]
Gallic acid	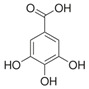	Antioxidant, anti-inflammatory, antimicrobial, and antiproliferative	[18,28]
Coumarin	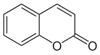	Antibiotic, bronchodilator, fungicide, anticoagulant, vasodilator, spasmolytic, and antithrombotic	[18,28]
Iriflophenone 3-C-glucoside	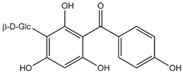	Antioxidant and antiproliferative	[30,31]
Antocyanin	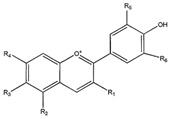	Antioxidant, anti-inflammatory, antidiabetic, and antiproliferative	[32,33]

## 2. Materials and Methods

This review involved two sections: the first was based on the application of *M. indica* by-products in the food industry. The second part involved a systematic review of the health effects of the mango plant and its derivatives. The strategies for searching and including clinical trials were based on the following aspects:

### 2.1. Focal Question

The focused question was “Can *M. indica* L. have beneficial effects on health?”.

### 2.2. Language

Only studies in English were selected. 

### 2.3. Databases

This review included studies in MEDLINE–PubMed, COCHRANE, EMBASE, Google Scholar, and Science Direct databases. The mesh terms used were *M. indica*, mango, mangiferin, human health, antioxidant, anti-inflammatory, obesity, diabetes, metabolic syndrome, and cardiovascular disease. 

The use of these descriptors helped identify studies related to mango and its health effects. We followed PRISMA (Preferred Reporting Items for a Systematic Review and Meta-Analysis) guidelines [34,35] to perform the search for clinical trials. Moreover, we also consulted in vivo and in vitro studies to help in the discussion section.

### 2.4. Study Selection

Conferences, abstracts, letters to editors, and other sources were evaluated but not included. The inclusion criteria were only human interventional studies, and the exclusion criteria were reviews, studies not in English, editorials, case reports, and poster presentations. 

### 2.5. Search and Selection of Relevant Articles

**Figure 2:**
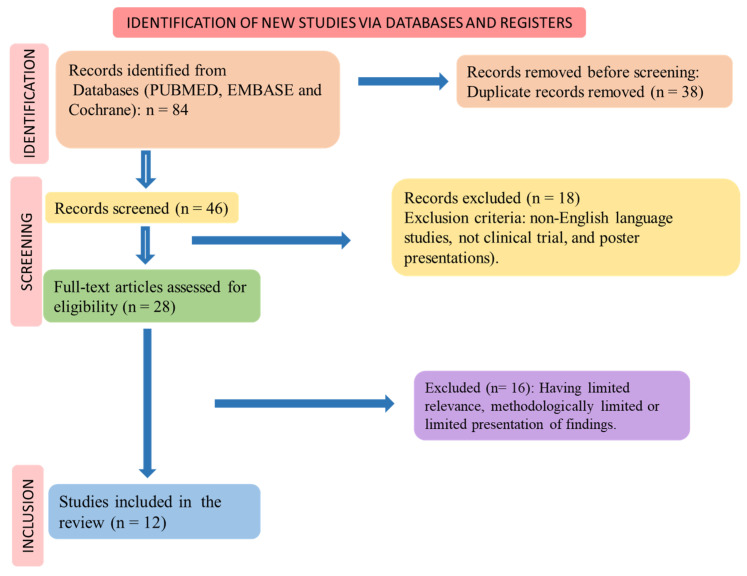
Flow diagram showing the study selection.

The PICO (Patients, Intervention, Comparison, and Outcomes) format was used to perform the systematic review, and the flow diagram shows the selection of the randomized clinical trials (Figure 2) and the inclusion and exclusion processes. 

### 2.6. Quality Assessment

We examined the Cochrane Handbook for the systematic review of interventions to evaluate the bias risks related to the selection of randomized clinical trials. 

## 3. Results

Figure 2 shows the study selection according to PRISMA (Preferred Reporting Items for a Systematic Review and Meta-Analysis) guidelines [34,35].

The selection of the included clinical trials is shown in Figure 2, and the results of the selected articles are found in Table 2. Twelve clinical trials were found. The risk of bias for these studies is shown in Table 3. Thirteen studies were included in this review. The findings of these studies revealed that the utilization of mango in different forms, including fresh mango slices, mango puree, mango by-products, mango leaf extract, fruit powder, and mangiferin yielded several positive effects. These effects encompassed improvements in glycemic control and plasma lipid levels, such as reductions in triglycerides, LDL cholesterol, and total cholesterol, as well as an elevation in HDL cholesterol. Additionally, mango consumption was associated with decreased appetite and food intake, enhanced mood scores, improved physical performance during exercise, enhanced endothelial function, and a reduced incidence of respiratory tract infections.

## 4. Discussion

### 4.1. M. indica By-Products

Mango by-products include bark, leaf, peel, and seed/kernel. For instance, about 15–25 million tons of mango peels are produced yearly, leading to harmful environmental effects. The phytocompounds found in these by-products can represent a fantastic source of bioactive compounds. 

Leaves and bark present almost 20 flavonoids and four new benzophenones. As examples, it is possible to cite mangiferin, gallic acid, ascorbic acid, quercetin-3-O-β-d glucoside, and protocatechuic acid, which play powerful antioxidant and anti-inflammatory roles [47]. Other bioactive compounds are functional lipids, unsaturated fatty acids, sterols, and soluble (starches and rhamno-galacturonans) and insoluble (hemicellulose and lignin) dietary fibers. The extract produced from the bark is traditionally used to treat hyperglycemia, anemia, diarrhea, cancer, and many other conditions [24,48,49,50]. The leaves are used in traditional medicine to treat hyperglycemia, obesity, dyslipidemia, cancer, and infectious and inflammatory conditions [12,51]. The most phenolic compounds in the leaves are mangiferin, quercetin, ellagic acid, and syringic acid. Peels are also a good mangiferin source; this compound can be considered heat-stable and pharmacologically active. The actions of mangiferin can include antiinflammation, antioxidant, anti-obesity, anti-type 2 diabetes, antitumor, and immunomodulatory effects [52,53]. 

Peels are a relevant source of dietary fiber (36–78 g/100 g of dry weight), and mango peel powder can be applied in the production of bakery and pasta products, beverages, snacks, ice cream, and meat products [54]. Peels also contain ascorbic acid, tocopherol, carotenoids, phenolic compounds, gallic acid, and derivatives [55,56,57,58]. Mango peel extracts play a significant role in exhibiting 2,2-diphenyl-1-picrylhydrazyl (DPPH) free-radical scavenging capacity and, consequently, can prevent or combat oxidative stress both in food products and in the human body. Polyphenols extracted from peels can be a natural substitute for artificial antioxidants on functional food or dietary supplements [59].

Kernels contain cellulose, starch, and pectin and can be used as an important dietary fiber source. The kernel phytocompounds include catechin, gallotannins, flavonoids, and benzophenones. Other phytocompounds are functional phytosterols, sesquiterpenoids, fatty acids, and tocopherols [48,55,56,60,61].

### 4.2. M. indica and Health Effects: Results from Clinical Trials

Table 2 summarizes the main findings of the included studies. Pinneo et al. [8] focused their research on analyzing the effect of mango consumption on biochemical parameters and satiety responses. However, the study has a limitation because it measures satiety hormones at only 45 min after consumption, as each hormone may have a different time curve. More frequent blood collections with a longer time window could provide a more accurate picture of the responses. 

Rosas et al. [36] found that consuming fresh mango significantly reduces blood glucose levels and LDL-c levels. This is a very interesting study; however, it has several limitations: the small sample size, the authors only used one dose of fresh mango, and the optimal dose cannot be determined.

According to Arshad et al. [37], the use of mango peel powder can improve lipid profiles in obese women. They also found important antioxidant activity, suggesting that mango peels have a considerable potential to control oxidative stress and dyslipidemia in obese individuals. Although they present promising results, there are important limitations: the small sample size and the inclusion of only women, which reduce the possibility of spoiling results.

Alkutbe et al. [38] studied the combination of nutrient extraction from two fruits, raspberry and passion fruit, combined with mango. They demonstrated that the combinations of mango/raspberry and mango/passion fruit can reduce the glycemic index in individuals with a healthy weight and in obese individuals. However, some limitations can be cited: the authors did not measure polyphenol content, including piceatannol (other authors have shown they can vary according to their country of origin) and factors such as insulin responses and changes in other hormones such as gastric inhibitory polypeptide (GIP) or glucagon-like peptide-1 (GLP-1). Only blood glucose responses after the intake of test meals were analyzed. Furthermore, healthy-weight individuals participated in only one of the two arms of the study. 

Anaya-Loyola et al. [39] found that by-products of mango juice (peel and pulp) did not prevent or eliminate upper respiratory and gastrointestinal infections. At the end of the study, the children still experienced some infection, but there was a low occurrence of gastrointestinal diseases and upper respiratory tract infection symptoms. Although the results are interesting, this study has some limitations: mango bioavailability was not evaluated, so the amount of bioavailable mango polyphenols is unclear; only children were included, so the findings may not be extended to other age groups; and the juice by-product was investigated in a single dose.

Martin-Rincon et al. [40] investigated supplementation with mango leaf extract (Zynamite^®^) in combination with quercetin and found that the polyphenols enhanced performance and muscle pain recovery, regardless of oral contraceptive intake. However, this study had limitations, such as the small sample size. Additionally, the diet during the assessment was not standardized, and the polyphenol content of the participants’ habitual diets had not been determined.

López-Ríos et al. [41] investigated the neurocognitive activity of mangiferin. The findings indicated significant spectral modifications in brain electrical activity. Although psychometric tests did not show significant modifications (except for reaction times across all groups), the study provides valuable evidence that mango leaf extract has no adverse effects on blood pressure, pulse, or heart rate variability. It is important to note that this study is limited in nature, being an exploratory investigation with a small number of participants.

Gelabert-Rebato et al. [42] showed the impact of mango leaf extract, rich in mangiferin, combined with luteolin on sprint exercise performance and muscle oxygen extraction capacity in active men. Although the results demonstrated promising effects of the extract on exercise performance, oxygen extraction, and cerebral oxygenation, the study was limited by its small sample size and the lack of an evaluation of oxidative stress biomarkers.

Buchwald-Werner et al. [43] conducted a study investigating the long-term consumption of a commercial powdered *M. indica* product (Careless™) on microcirculation and glucose metabolism. The researchers deemed these findings promising, indicating a modest beneficial effect of *M. indica* fruit preparation on microcirculation, endothelial function, and glucose metabolism by the end of the study. Some limitations can be cited for this study. The age range of the patients included was wide; the table with socio-demographic data does not make it clear how many men and women took part in the study.

Gerstgrasser et al. [44] observed the effects of Careless™ (mangiferin) on microcirculation. The results showed promising and beneficial effects in healthy women. These findings suggest that Careless™ may have potential therapeutic applications for individuals with microcirculation alterations and endothelial dysfunction. However, due to the small sample size, these findings cannot be extended to a larger number of patients.

In a study conducted by Na et al. [45], the effects of mangiferin on serum lipid profiles and free fatty acid concentrations were examined in overweight and dyslipidemic patients. Their findings suggest that mangiferin may improve lipid metabolism in individuals with these conditions. However, due to the broad age range included in the study, the findings may have limitations.

Patnaik [46] observed the effectiveness of mango leaves in controlling diabetes. Although this study is interesting, some limitations must be noted. Firstly, a questionnaire was used to evaluate the patients’ anthropometric and biochemical parameters, limiting the reliability of the results. Furthermore, the sample was small (30 patients divided into three groups).

### 4.3. Antioxidant Effects of M. indica L.

Oxidative stress is related to the development or worsening of different metabolic conditions such as obesity, hyperglycemia, dyslipidemia, and cardiovascular disease. When considering *M. indica* L.’s *antioxidant properties*, it is essential to emphasize its phytochemical compounds [62]. The most relevant polyphenols with antioxidant activity in the mango fruit are the class of flavonoids (catechins, quercetin, kaempferol, rhamnetin, anthocyanins, and tannic acid) and the class of xanthones, which includes mangiferin (C2-b-d-glucopyranosyl-1,3,6,7-tetrahydroxyxanthone) [63]. Mangiferin can be obtained from the roots, bark, leaves, and fruits of *M. indica* L. and presents anticancer, antimicrobial, antiatherosclerotic, antiallergenic, anti-inflammatory, high iron-chelating, analgesic, and immunomodulatory properties as well as the capacity to reduce ROS production [62,64].

Moreover, the administration of mangiferin to diabetic nephropathy rats reduced serum levels of advanced glycation end products and enhanced antioxidant enzymes, including superoxide dismutase and glutathione peroxidase [65]. Also, another study involving the use of mangiferin in diabetic rats identified increased liver levels of the following antioxidant enzymes: superoxide dismutase, catalase, and glutathione peroxidase [66].

Furthermore, according to a study developed by Sferrazzo et al. [67], *M. indica* L. has proven to be a great example of an antioxidant source that stabilizes free radicals, contributing to minimizing oxidative stress. This experimental analysis using mango leaf extract (MLE) obtained from Sicilian mango (Lentini, Italy) has shown that the polyphenol derivatives of that extract present antioxidant activity. This activity was confirmed by using MLE treatment in humans, reducing the gene expression of pro-inflammatory enzymes and cytokines such as IL-1β, IL-6, TNFα, and COX2.

**Figure 3:**
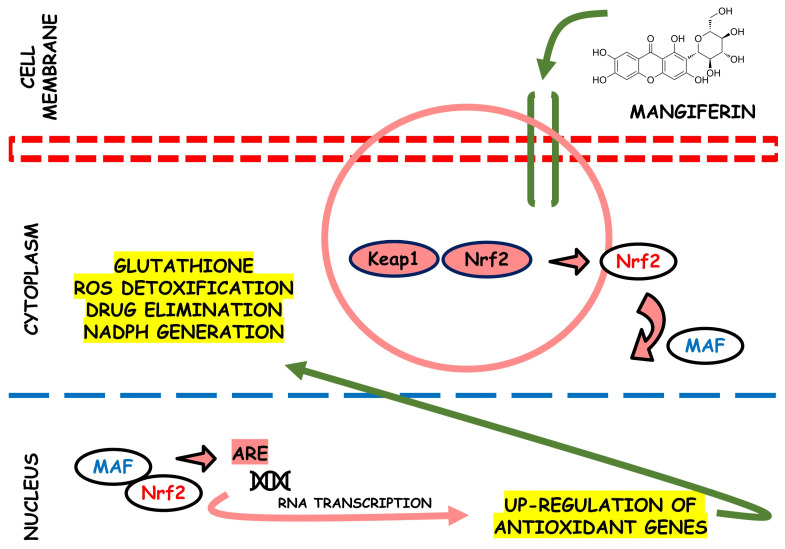
This scheme illustrates the potential antioxidant efficacy of mangiferin. This bioactive agent shows its effectiveness by potentially interacting with the nuclear factor erythroid 2-related factor 2 (Nrf2) pathway. This interaction initiates a series of events that activate cellular defense mechanisms against oxidative stress. The multifaceted actions of mangiferin in scavenging reactive oxygen species and enhancing endogenous antioxidant systems make it a compelling candidate for therapeutic interventions in oxidative stress-related disorders.

In rats, a study identified the antioxidant activity of the MLE on several oxidative stress systems induced by H_2_O_2_ related to antispasmodic activity spasmolytic. The results showed that chronic intake with MLE reduced lipid peroxidation in the small intestine and counteracted the ROS effects, protecting tissues from oxidative damage [68]. Figure 3 provides an illustration depicting the potential antioxidant effects of mangiferin. This bioactive compound may plausibly interact with Nrf2, as supported by prior research findings [69].

### 4.4. Anti-Inflammatory Effects of M. indica L.

As an oxidative stress, inflammation is related to obesity, hyperglycemia, dyslipidemia, metabolic syndrome, and cardiovascular diseases. As mango fruit and by-products possess many bioactive compounds, they can be considered to prevent inflammatory processes. A study developed by Sferrazzo et al. (2022) evaluated the anti-inflammatory activity of mango leaf extract (MLE) using macrophages treated with lipopolysaccharide (LPS) as an in vitro model. The use of LPS caused an increase in pro-inflammatory genes such as cyclooxygenase-2 (COX-2), interleukin-6 (IL6), interleukin-1β (IL-1β), and tumor necrosis factor-α (TNF-α), which was downregulated to regular levels by the use of MLE. The extract also caused a reduction in pro-inflammatory cytokines genes, IL-1β, IL-6, and COX-2 in hepatic stellate cells treated with LPS [67].

Mango pulp is an excellent source of polyphenols, most of them derived from gallic acid and galloyl-polyphenols such as mono-galloyl glucose and gallotannins (hexa-to nona-*O*-galloyl-glucoses). The anti-inflammatory properties of gallotannins and their associated metabolites (such as gallic acid and 4-O-methylgallic acid) are related to the reduction of pro-inflammatory cytokines, intracellular adhesion molecule 1 (ICAM-1), nuclear factor kappa B (NF-κB), and vascular cell adhesion molecule 1 (VCAM-1). Also, gallic acid can reduce inflammation responses in intestinal epithelial cells by reducing pro-inflammatory cytokines such as IL-1, IL-6, IL-17, and TNF-α and inducing anti-inflammatory cytokines such as IL-4 and IL-10. In addition, mango stem bark extract also showed anti-inflammatory activity by inhibiting TNF, prostaglandin E2 (PGE2), and nitric oxide (NO) [70]. 

**Figure 4:**
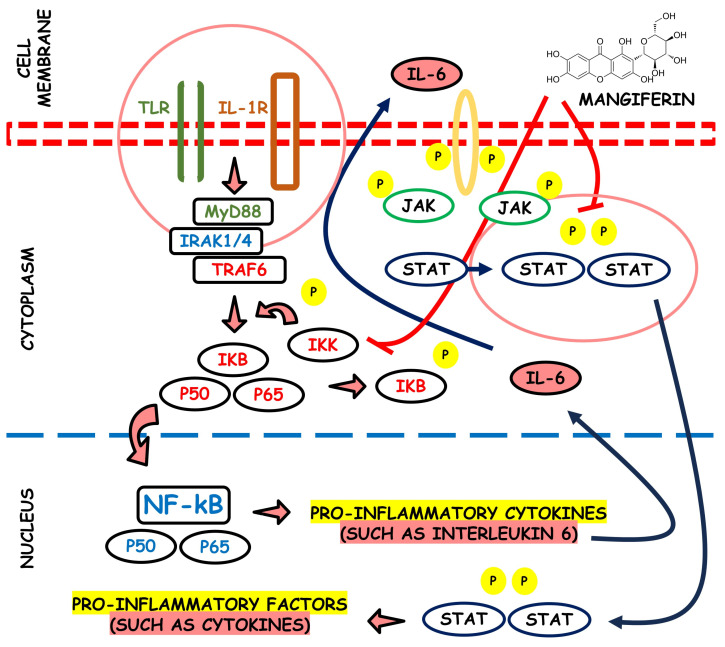
Visual representation of the potential anti-inflammatory attributes linked with the compound mangiferin.

Phospholipases A2 (PLA2) are a family of enzymes that regulate the arachidonic acid pathway; this, by the action of cyclooxygenase and lipoxygenase, liberates pro-inflammatory mediators such as leukotrienes, thromboxanes, prostacyclins, and prostaglandins. It has been proved that the aqueous stem bark extract of *M. indica* L. can suppress inflammation processes by inhibiting inflammatory PLA2 belonging to group IA, i.e., NN-XIa-PLA2 (a purified PLA2 enzyme obtained from *Naja naja* venom). The extract inhibited both in vitro and in situs PLA2 activity and dose-dependently inhibited the in vivo edema-inducing activity of NN-XIa-PLA2. Also, oral extract administration reduced mice ear edema induced by arachidonic acid and phorbol myristate acetate [71]. Figure 4 depicts the potential anti-inflammatory properties associated with mangiferin.

### 4.5. Metabolic Effects of M. indica L. 

Diabetes is considered one of the leading causes of death worldwide. Mango by-product consumption has potential value in treating risk factors for diabetes [21,25,72,73]. A study showed the hypoglycemic effect of the MLE in male normoglycemic and diabetic rats and observed a significant decrease in glycemia, surpassing the effects of glibenclamide. The extract maintained long-term hypoglycemic actions effectively and significantly improved insulin sensitivity in an animal model.

A study performed with human HepG2 and HL-7702 hepatocytes was evaluated under a high glucose level and submitted a mangiferin derivative compound: 1,3,6,7-tetrapropylene acyloxy-ketone (TPX). The results showed augmented glycogen synthesis and inhibition of gluconeogenesis. This evaluation of the article occurs due to the regulation of GSK3β, G6Pase, and PEPCK. Energy homeostasis and hepatic insulin resistance were also improved due to actions in AMPK and PI3K/AKT. These results are promising for the treatment of insulin-resistance-related diseases, including metabolic-associated fat liver diseases and diabetes [74].

Noh et al. [75] conducted a study examining the effects of mangiferin on glycemia and obesity both computationally and in C57BL/6 mice subjected to a high-fat diet. Their in silico model revealed mangiferin’s capacity to bind with inflammatory biomarkers associated with macrophages and autophagy proteins. In vivo experiments demonstrated significant reductions in body weight and glucose and lipid metabolism improvements, as well as decreased insulin resistance in obese subjects. Furthermore, mangiferin decreased Kupffer cells and macrophages within adipose tissue and inhibited TNF-α and NF-κB expression in the same tissue. Additionally, it enhanced the liver’s expression of fibroblast growth factor 21. In a separate investigation, Apontes et al. [76] administered 400 mg of mangiferin (derived from bark extract) per kilogram of diet to mice for five weeks. The outcomes demonstrated heightened glucose and pyruvate oxidation and increased ATP production without impacting fatty acid oxidation. This redirection of fuel utilization appeared to safeguard carbohydrates. The authors speculated that mangiferin has the potential to enhance carbohydrate metabolism. Another study by Saleem et al. [77] explored the effectiveness of mango leaf extract in diabetic Swiss albino mice. Their findings exhibited lowered postprandial glycemia and an improved lipid profile.

Irondi et al. [78] examined the impact of *M. indica* seed flour on diabetic Wistar rats subjected to a high-fat diet. Following this, the rats were provided diets enriched with flour or were administered metformin for 21 days. The inclusion of the flour led to enhancements in fasting glycemia, liver glycogen levels, glycosylated hemoglobin, and lipid profile, as well as reductions in hepatic and pancreatic malondialdehyde. Additionally, it resulted in favorable alterations in markers of liver function, including plasma aspartate aminotransferase, alanine aminotransferase, and alkaline phosphatase levels in diabetic rats.

Besides diabetes, the global prevalence of obesity has been a growing concern for public health officials worldwide. Obesity is a complex and multifactorial condition influenced by genetics, environment, lifestyle, and socioeconomic factors. The prevalence of obesity is indeed particularly high (>25% of adults) in several regions, including the Americas, the Middle East, and Pacific Island communities [79].

Some signaling pathways contribute to pro-obesity and anti-obesity mechanisms within the body. Pro-obesity mechanisms encompass insulin resistance, inflammation in adipose tissue, and the process of adipogenesis. Conversely, anti-obesity mechanisms involve signaling pathways and processes that aim to counteract these pro-obesity mechanisms [80,81]. In this way, Taing et al. [82] and Fang et al. [81] concluded that extracts from *M. indica* peel effectively hinder adipogenesis in vitro in a dose-dependent manner by suppressing mitotic clonal expansion, genes linked to mitochondrial biogenesis, and fatty acid oxidation in 3T3-L1 adipocytes via AMP-activated protein kinase (AMPK) signaling. 

In addition to obesity, dyslipidemia frequently co-occurs, raising concerns about its potential implications, notably in terms of cardiovascular diseases, which continue to be the leading cause of global mortality [83]. Several studies have been conducted on mangoes to investigate their effects on cholesterol profiles and their potential relevance to cardiovascular health.

Various compounds present in *M. indica* leaves may have a twofold impact: firstly, by blocking enzymes responsible for the de novo synthesis of cholesterol, and secondly, by competing with cholesterol absorption [84]. Furthermore, in male Wistar rats, a standardized mango leaf extract demonstrated a more significant cholesterol-reducing effect compared to atorvastatin. This effect was attributed to three key molecules: iriflophenone 3-C-β-d-glucoside, mangiferin, and 3β-taraxerol [85]. In hyperlipidemic rats, a methanolic extract from mango leaves containing a significant amount of mangiferin with a bioaccessibility of 12% successfully decreased blood lipid levels [86].

In another study, Wistar rats underwent ischemia injury followed by reperfusion. The control group exhibited significant cardiac dysfunction, elevated serum cardiac injury markers, increased lipid peroxidation, and a notable decrease in tissue antioxidants. However, pretreatment with mangiferin effectively restored the balance between oxidants and antioxidants in the heart tissue, preserved cell membrane integrity, and reduced levels of pro-inflammatory cytokines, pro-apoptotic proteins, and transforming growth factor beta (TGF-β). Additionally, mangiferin significantly reduced the phosphorylation of p38 and c-Jun N-terminal protein kinase (JNK) while enhancing the phosphorylation of extracellular signal-regulated protein kinases 1 and 2 (ERK1/2), indicating its role in modulating the mitogen-activated protein kinase (MAPK) signaling pathway [87].

From the perspective of microcirculation, *M. indica* fruit preparation (Careless™) was administered in healthy women. It triggered the activation of evolutionarily conserved metabolic sensors, namely sirtuin 1 and adenosine monophosphate-activated protein kinase. These sensors have been recognized as pivotal in regulating microcirculation and maintaining endothelial function. At the same time, an in vitro test in primary human umbilical vein endothelial cells promoted endothelial nitric oxide synthase activation [44].

## 5. Conclusions

Incorporating mango into one’s diet has been shown to enhance glycemic control, regulate plasma lipid levels, increase satiety, and improve endothelial function. The collective findings from the included studies indicate that the utilization of mango in various forms—ranging from fresh mango slices and mango puree to mango by-products, mango leaf extract, fruit powder, and mangiferin—yield many favorable effects. These encompass enhancements in glycemic control and improvements in plasma lipid profiles, including reductions in triglycerides, LDL cholesterol, and total cholesterol, alongside increased HDL cholesterol levels. Additionally, mango consumption is correlated with diminished appetite and reduced food intake, elevated mood scores, augmented physical performance during exercise, improved endothelial function, and a decreased incidence of respiratory tract infections. Despite the health benefits, further standardized clinical trials are imperative to establish the optimal administration, dosage, and intervention duration for mango and its derivative by-products. It is also crucial to investigate further the potential risks associated with their use.

The utilization of mango by-products aligns with the current trend and expanding market demand for improved, healthier products. Nutraceutical items could be more affordable, making them accessible to low-income populations. Additionally, this approach contributes to reducing environmental harm, a critical factor in ensuring humanity’s sustainable presence on the Earth.

In light of this current landscape and alignment with the Sustainable Development Goals, there is a pressing need to promote the active development of nanomedicines derived from mangoes. This innovative approach holds tremendous potential for addressing various healthcare challenges. We can pioneer groundbreaking therapeutic approaches while safeguarding the environment by harnessing the distinctive attributes of mango-derived compounds at the nanoscale.

## Figures and Tables

**Table 2:** Clinical trials showing the effects of *M. indica* L. or its derivatives on human health.

Study Type and Patients	Interventions	Results	Side Effects	Ref.
Interventional study; 23 overweight and obese adults, including 15 men and 8 women, 18–55 y; San Diego, United States.	Participants received 100 kcal of mango snacks	After 45 min of ingestion, significant ↑ in cholecystokinin (*p* ≤ 0.05) and adiponectin (*p* ≤ 0.05) levels. Additionally, participants reported ↓ hunger and an increase in feelings of satiety. Interestingly, no significant differences were found in the levels of satiety hormones, including leptin, ghrelin, and peptide YY, among the interventions. The consumption improved postprandial glycemic responses.	Not mentioned by the authors.	[8]
Randomized crossover clinical trial; 27 overweight/obese patients (16 men and 11 women) without gluten allergies, non-smokers, non-pregnant, and non-lactating; United States.	The individuals were divided and analyzed in two phases: for 12 weeks, they were offered 100 Kcal of fresh mango/day; for another 12 weeks, they were given 24 g of LFC/day.	Fresh mango: ↓ glycemia (*p* = 0.004) in weeks 4–12. There were no significant modifications in insulin levels. ↓ LDL-c, and additionally a slight increase in adiponectin. ↓ AST and PCR and ↑ total antioxidant capacity. LFC: ↑ TG levels and basal insulin, ↓ adiponectin at the end of the 12 weeks. ↑ PCR but no significant change in total antioxidant capacity. For both interventions, there were no significant differences in HbA1C values, anthropometry, or blood pressure.	Not mentioned by the authors.	[36]
Interventional trial; 31 overweight women, 25–45 y (no history of diabetes, hypertension, cardiovascular, or liver diseases); Pakistan.	Over a period of 84 days, 21 subjects were administered 1 g of mango peel powder 2x/d, 30 min before each meal. 10 subjects received no treatment.	The mango group showed a decrease in weight gain and an increase in antioxidant levels. ↓ Urea, creatinin, TG, TC, and LDL-c. ↑ HDL-c levels when compared to the control group. The mango group also experienced a decrease in appetite compared to the control group.	Not mentioned by the authors.	[37]
39 participants: group 1/healthy weight was divided into two subgroups: one subgroup consumed a combination of raspberry and mango, and the other subgroup consumed a combination of passion fruit and mango. Group 2/obesity completed both subgroups; United Kingdom.	Group A received 162 g of raspberry and 114.2 g of mango in each meal, while group B received 150 g of passion fruit and 114.2 g of mango in each meal.	The average glycemic index for raspberry/mango was higher in the healthy weight group compared to the obesity group. The blood glucose levels of participants with a healthy weight were significantly ↑ after consuming raspberry and mango compared to the non-energy meal. The blood glucose levels in the obese group were significantly ↑ throughout the raspberry/mango arm at 15 and 120 min postprandial compared to the NE meal. ↓ postprandial blood glucose levels when compared to consuming the whole fruit.	Not mentioned by the authors.	[38]
Randomized, double-blind, parallel-group trial with controlled intervention; 65 patients, 6–8 y (upper respiratory and gastrointestinal tract infections); Mexico.	The treated group received 2 g of mango by-product (peel and pulp) mixed in 50 mL of water, while the control group received flavored water daily for a period of 2 months.	↓ incidence of diarrhea, nasal congestion, dry cough, gastrointestinal tract infections, and abdominal inflammation. The JBP group showed ↑ counts of red blood cells, hemoglobin, hematocrit, platelets, and neutrophils compared to the control group. The control group experienced ↑ lymphocyte count compared to the treated group.	Not mentioned by the authors.	[39]
Double-blind trial; 48 healthy participants, 18 men and 30 women, 18–45 y; Spain.	1 h before running and every 8 h/24 h (after running), the participants ingested either a placebo or 140 mg of Zynamite^®^ (mangiferin-rich mango leaf extract) with 140 mg of quercetin.	A single dose of 140 mg of Zynamite combined with a similar amount of quercetin attenuated pain and muscle damage caused by running and accelerated the recovery of muscle performance. This analgesic effect may have been mediated by the free-radical scavenging properties of Zynamite and quercetin, since free radicals have been implicated in nociception.	Not mentioned by the authors.	[40]
Two double-blind, placebo-controlled, randomized crossover clinical trial; 38 participants, including 16 men and 16 women, 18–40 y; South Africa.	The participants received 500 mg of MLE at either 60 min (study 1) or 90 min (study 2).	In the first study, MLE improved all mood state profile scores, with a significant improvement in the “fatigue” score. In the second study, there was a trend toward faster reaction time in the MLE group compared to the placebo. The Profile of Mood States (POMS) score for “depression” improved in the caffeine group. In both studies, MLE caused significant changes in cortical brain electrical activity during cognitive challenges, distinct from the attenuated spectral changes induced by caffeine.	Not mentioned by the authors.	[41]
Double-blind, crossover, counterbalanced design; 12 healthy and physically active men, 21.3 ± 2.1 y; Spain.	The placebo group received capsules containing 500 mg of maltodextrin, while the treatment group received capsules containing 50 mg and 100 mg of luteolin, along with 100 mg and 300 mg of mangiferin, for 15 days.	The supplementation improved exercise performance, facilitated muscular oxygen extraction, and improved cerebral oxygenation without increasing VO2 compared to the placebo.	Not mentioned by the authors.	[42]
Monocentric, randomized, placebo-controlled, 3-armed, double-blind trial with a parallel design; 75 healthy participants, 42 men and 33 women, 40–70 y, non-smokers; Switzerland.	The participants were assigned to three groups: the first group received 100 mg of a 100% mango fruit powder, the second group received 300 mg of the fruit powder, and the third group received a placebo/4 weeks.	The reactive hyperemic microcirculatory flow increased, particularly in the 100 mg group. The group that consumed 300 mg showed ↓ postprandial blood glucose compared to the placebo. The ingestion of 300 mg significantly improved postprandial endothelial function in individuals with impaired endothelial function following high-dose glucose ingestion.	Not mentioned by the authors.	[43]
Monocentric, randomized, double-blind, crossover trial; 10 healthy women, 40–70 y; United States.	The participants consumed 100 mg followed by 300 mg or 300 mg followed by 100 mg of Careless™ (mangiferin) in a single dose.	After 6 h: ↑ cutaneous blood flow over time with both 100 mg (54% above baseline, *p* = 0.0157) and 300 mg (35% above baseline, *p* = 0.209) of Careless™. The reactive hyperemia response was slightly improved 3 h after ingestion compared to the pre-test with 30 mg of Careless™.	Not mentioned by the authors.	[44]
Randomized, double-blind, placebo-controlled trial; 97 overweight patients with hyperlipidemia; 52.1 ± 7.2 y; China.	The subjects consumed a daily dose of 150 mg of mangiferin or a placebo/12 weeks.	↓ TG and FFA in the mangiferin supplementation group. ↓ HOMA-IR and total serum fatty acids, SFA, MUFA, PUFA, total serum n-3 fatty acids, and total serum n-6 fatty acids compared to the control group. ↑ HDL-c, L-carnitine, β-hydroxybutyrate, and acetoacetate levels.	No side effects or changes in liver enzymes or renal variables were observed.	[45]
Clinical trial; 30 patients (gender not specified; 30–69 y); India.	Group A followed diet and medication; group B: diet, medication, and mango-leaf powder (1–2 teaspoons in 150 mL of water, taken 2x/d on an empty stomach); and Group C: diet, medication, and a placebo.	After approximately 1 month, both group A and group B showed significant reductions in both fasting and postprandial blood sugar levels. However, group C showed only a slight reduction in blood sugar levels.	Not mentioned by the authors.	[46]

Abbreviations: ↑: increase; ↓: decrease; AST: aspartate aminotransferase; FFA: free fatty acids; HbA1C: glycated hemoglobina; HDL-c: high-density lipoprotein cholesterol; HOMA-IR: homeostatic model assessment of insulin resistance; JBP: mango juice by-product; LDL-c: low-density lipoprotein cholesterol; LFC: low-fat cookies; MLE: mango leaf extract; MUFA: monounsaturated fatty acids; NE: nutrient extracted fruit; PCR: c-reactive protein; PUFA: polyunsaturated fatty acids; SFA: saturated fatty acids; TC: total cholesterol; TG: triglycerides; VO2: oxygen consumption.

**Table 3:** Descriptive table of the biases in the included randomized clinical trials.

Study	Question Focus	Appropriate Randomization	Allocation Blinding	Double Blind	Losses (<20%)	Prognostics or Demographic Characteristics	Outcomes	Intention to Treat Analysis	Sample Calculation	Adequate Follow-Up
[8]	Yes	No	Yes	No	Yes	No	Yes	Yes	No	Yes
[36]	Yes	Yes	Yes	No	Yes	No	Yes	Yes	No	Yes
[37]	Yes	Yes	Yes	No	Yes	No	Yes	Yes	No	Yes
[38]	Yes	Yes	Yes	No	Yes	No	Yes	Yes	No	Yes
[39]	Yes	No	Yes	Yes	Yes	No	Yes	Yes	No	Yes
[40]	Yes	No	Yes	Yes	Yes	No	Yes	Yes	No	Yes
[41]	Yes	Yes	Yes	No	Yes	No	Yes	Yes	No	Yes
[42]	Yes	No	Yes	Yes	Yes	No	Yes	Yes	No	Yes
[43]	Yes	Yes	Yes	Yes	Yes	No	Yes	Yes	No	Yes
[44]	Yes	Yes	Yes	Yes	Yes	No	Yes	Yes	No	Yes
[45]	Yes	Yes	Yes	Yes	Yes	No	Yes	Yes	No	Yes
[46]	Yes	No	Yes	No	Yes	No	Yes	Yes	No	Yes

## Data Availability

Not applicable.
